# Relationship between Neovascular Density in Swept Source-Optical Coherence Tomography Angiography and Signs of Activity in Exudative Age-Related Macular Degeneration

**DOI:** 10.1155/2019/4806061

**Published:** 2019-07-09

**Authors:** Martin Stattin, Julia Forster, Ahmed Daniel, Alexandra Graf, Katharina Krepler, Siamak Ansari-Shahrezaei

**Affiliations:** ^1^Karl Landsteiner Institute for Retinal Research and Imaging, Vienna, Austria; ^2^Department of Ophthalmology, Rudolf Foundation Hospital, Vienna 1030, Juchgasse 25, Austria; ^3^Center for Medical Statistic, Informatics, and Intelligent Systems, Medical University of Vienna, Vienna 1090, Spitalgasse 23, Austria; ^4^Department of Ophthalmology, Medical University of Graz, Graz 8036, Auenbruggerplatz 1, Austria

## Abstract

**Purpose:**

To assess the relationship between signs of activity in exudative neovascular age-related macular degeneration (nAMD) following anti-vascular endothelial growth factor (anti-VEGF) treatment and morphology of choroidal neovascularization (CNV) based on neovascular density as imaged using swept source-optical coherence tomography angiography (SS-OCTA) in a qualitative manner.

**Methods:**

A single-cohort retrospective data analysis from one tertiary eye care center. Seventy-seven eyes of 72 patients were included and their charts reviewed which had been started on intravitreal injections with anti-VEGF for nAMD at least one year prior to enrollment. Clinically active disease was evaluated by slit-lamp fundus examination and spectral domain-OCT B-scans. Morphological appearance in SS-OCTA was characterized based on 5 different criteria and subsequently divided into 3 groups: predominantly hyperdense, minimally hyperdense, and hypodense lesions.

**Results:**

Fifty-eight eyes (75%) were considered clinically active and 19 eyes (25%) clinically inactive. CNV was depicted in 71 eyes (92%) by SS-OCTA and separated accordingly into predominantly hyperdense (32%), minimally hyperdense (34%), and hypodense lesions (34%). A borderline significant difference in the probability of neovascular activity for predominantly hyperdense lesions compared to hypodense lesions was detected (*p*=0.05).

**Conclusions:**

Hyperdense choroidal neovascularization based on qualitative assessment of flow density showed a significant relation to active disease. Inactivity could not be matched adequately. This study demonstrated the potential usefulness of SS-OCTA for guidance of treatment in age-related macular degeneration.

## 1. Introduction

To date, dilated fundus examination, fluorescein angiography (FA), indocyanine green angiography (ICGA), and optical coherence tomography (OCT) were generally employed for the diagnosis, classification, and therapeutic guidance of age-related macular degeneration (AMD) complicated by choroidal neovascularization (CNV) [1].

OCT angiography (OCTA) is a relatively new noninvasive imaging modality, based on high-frequency scanning for the detection of red blood cell movement [2]. This novel technique provides the potential to visualize depth-resolved blood flow in the retinal microcirculation without the risk of dye-associated adverse events such as nausea or anaphylaxis caused by FA/ICGA [3]. Furthermore, OCTA can separately detect the superficial vascular and the deep vascular networks that are generally overlaid and appear fused in standard angiographies [4]. However, the utility of OCTA alone in the detection and observation of neovascular (n)AMD is still under debate [5]. Previous studies have demonstrated the superiority of FA/ICGA to OCTA concerning sensitivity and specificity in the diagnosis of untreated CNV [6, 7]. Nevertheless, the isolation of CNV morphology and its repeatability in OCTA scans indicate the potential capacity of this method in the qualitative assessment and monitoring of the response to antiangiogenic treatment in this chronic disease [8–10].

The purpose of this study was to evaluate a new qualitative CNV classification based on neovascular density imaged by swept source (SS)-OCTA and to assess its relevance regarding clinical activity. A significant relationship between CNV morphology and nAMD subsequent to anti-vascular endothelial growth factor (anti-VEGF) treatment would benefit daily routine and ease decision-making.

## 2. Materials and Methods

### 2.1. Patients

Consenting patients who had commenced treatment with intravitreal injections (IVIs) of anti-VEGF for nAMD that was initially diagnosed by fluorescein angiography ≥12 months prior to enrollment and who were followed up from March to July 2017 at our tertiary retina center (Medical Retina Unit, Department of Ophthalmology; Rudolf Foundation Hospital Vienna; Karl Landsteiner Institute for Retinal Research and Imaging) additionally underwent OCTA imaging at the time of recruitment. The study protocol adhered to the tenets of the Declaration of Helsinki. Standard follow-up included best-corrected visual acuity (BCVA) measurement using early treatment diabetic retinopathy study (ETDRS, 4 m) charts, dilated slit-lamp fundus examination (Haag-Streit AG, Bern, Switzerland) by a senior medical retina specialist and imaging with spectral domain (SD)-OCT (Zeiss Cirrus HD 4000, Carl Zeiss Meditec AG, Jena, Germany). Inactive disease was defined as (1) absence of fluid (intraretinal, subretinal or subretinal pigment epithelium) on SD-OCT B-scans, (2) no new macular hemorrhage on biomicroscopy, and (3) no intravitreal treatment with anti-VEGF within the past 9 months. If all criteria applied to an eye, the lesion was considered as inactive.

### 2.2. Imaging

SD-OCT B-scans were acquired using the central 1 mm (500 *μ*m radius) zone for accurate centration within the ETDRS grid. The scan was excluded from the analysis if adjustment of these parameters was not possible due to severe distortion of the retinal architecture or motion artefacts due to absent fixation.

Consenting patients were examined with a beta-version of Topcon's DRI Triton SS-OCTA (Topcon Corporation, Tokyo, Japan) device operating at 100,000 A scans per s on 1 mW input power. One notable feature is its OCTA Ratio Analysis, a full-spectrum algorithm that relies on the detection of motion contrast to create a decorrelation signal. Topcon's OCTA analysis software (IMAGEnet 6) provided three-dimensional (3D) 3 × 3 mm, 4.5 × 4.5 mm, 6 × 6 mm, and even 9 × 9 mm macular cubes with automated subdivision into four different en-face segments: the superficial inner retinal plexus, the deep inner retinal plexus, the outer retina, and the choriocapillaris. The segments were concordantly matched with SS-OCT B-scans to visualize the layer of the neovascular complex layer. Each B-scan consisted of 320 A scans and each volume (3 × 3 mm, 4.5 × 4.5 mm, 6 × 6 mm) consisted of 320 B-scans, respectively, of 512 B-scans (9 × 9 mm). The pixel size ranged from 9.4 to 18.8 *μ*m transversal resolutions according to the selected cube. The digital axial resolution was 2.6 *μ*m. Acquired volumes in 4.5 × 4.5 mm cubes as standard of care were generated for each eye by orthogonal registration centered at the macula and augmented by additional sizes. If more than one cube size was applied, the scan with the highest resolution (i.e., the smallest possible cube size) imaging the full lesion was analyzed. The integrated artefact removal option for projection artefacts was applied to the choriocapillaris and the outer retina slab. The integrated software was used to delineate the outer retina and choriocapillaris segments. Automated segmentation lines were manually altered and moved progressively in both directions by a skilled resident to ensure visualization of all circumscribed CNV borders.

### 2.3. Grading

A medical retina fellow, who was blinded to the funduscopy and Zeiss' SD-OCT B-scans, had access to the entire volumetric dataset and identified neovascular networks by observing multiple 2D en-face scans and categorized them into three groups based on the presence or absence of five established morphological criteria:

(1) Numerous branching capillaries between major vessels separating the lesion area into fractals, (2) end-to-end anastomoses or intervascular anastomoses within the lesion, (3) arcades or vascular loops at the vessel termini, (4) major, well-defined filamentous vessels, and (5) peri- or intralesional nonvascularized hypointense halos surrounding or embedding the CNV membrane only if masking by blood, fluid, pigment, or thresholding could be excluded [11].

**Figure 1:**
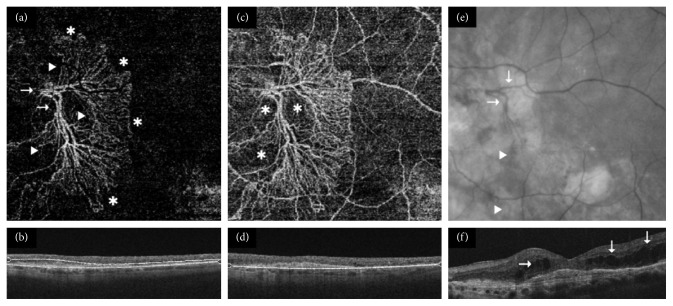
Clinically active predominantly hyperdense choroidal neovascularization (CNV). (a) A sea-fan-like CNV formation with major feeder vessels (arrows) but mostly capillary branching (arrow-heads) and peripheral arcades or loops (^*∗*^) in (b) a nonperfused outer retina 6 × 6 mm swept source-optical coherence tomography angiography (SS-OCTA) segmentation image (DRI Triton, Topcon). (c) The same en-face layer revealed nonvascularized halos (^*∗*^) within the lesion and helped to localize its borders within (d) an underlying 6 × 6 mm choriocapillaris slab. (e) The mature vasculature was well visible in the corresponding infrared fundus photography (arrows). Smaller loops and branches (arrow-heads) could also be identified. (f) The spectral domain-OCT B-scan (Cirrus HD 4000, Zeiss) was acquired at the time of the last intravitreal injection 3 months prior to the SS-OCTA scan and demonstrated extensive intraretinal fluid (arrows).

**Figure 2:**
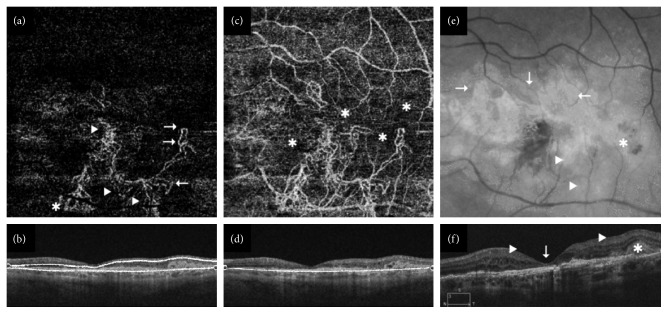
Clinically active minimally hyperdense choroidal neovascularization (CNV). (a) The 6 × 6 mm swept source-optical coherence tomography angiography (SS-OCTA) C-scan (DRI Triton, Topcon) displayed extensive CNV formation in the macular region with areas of peripheral arcades and loops (arrows), capillary branching (arrow-heads), and sea-fan-shaped peripheral anastomoses (^*∗*^) corresponding to (b) the otherwise nonperfused outer retina segmentation image. (c) The choriocapillaris layer highlighted the multitudinous nonvascularized halos of capillary dropout (^*∗*^) within the lesion area in addition to some projection artefacts in (d) an OCT B-scan segmentation image. (e) The infrared fundus photography revealed subretinal fibrotic tissue around the fovea (arrows) in addition to major neovascular vessels (arrow-heads) and temporally sprayed hemorrhage (^*∗*^). (f) A macula with enlarged foveal depression (arrow), intraretinal fluid (arrow-heads), and underlying fibrosis next to prominent subretinal hyperreflective material (^*∗*^) were depicted in the routine spectral domain-OCT B-scan (Cirrus HD 4000, Zeiss).

**Figure 3:**
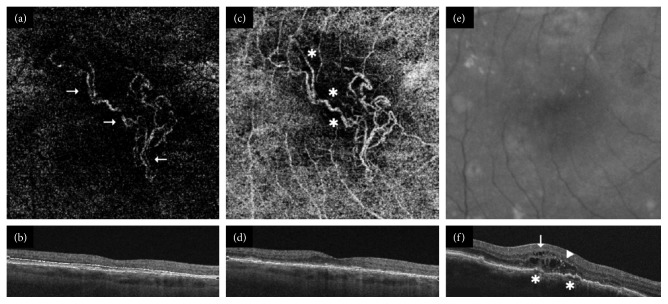
Clinically active hypodense choroidal neovascularization. (a) Numerous well-defined vessels (arrows) without capillaries, small branches, or terminal loops were depicted by Topcon's DRI Triton swept source-optical coherence tomography angiography (SS-OCTA) in a 4.5 × 4.5 mm en-face cube corresponding to (b) an outer retina B-scan segmentation image. (c) The same lesion was highlighted by an extensive nonvascularized halo (^*∗*^) in (d) a 4.5 × 4.5 mm choriocapillaris B-scan. (e) No neovascular vessels nor evidence of clinical activity was visible on infrared fundus photography of the same macular region. (f) Intraretinal fluid (arrows) and hyperreflective dots (arrow-heads) as well as an irregular retinal pigment epithelium detachment (^*∗*^) were observed in the spectral domain-OCT B-scan (Cirrus HD 4000, Zeiss).

**Figure 4:**
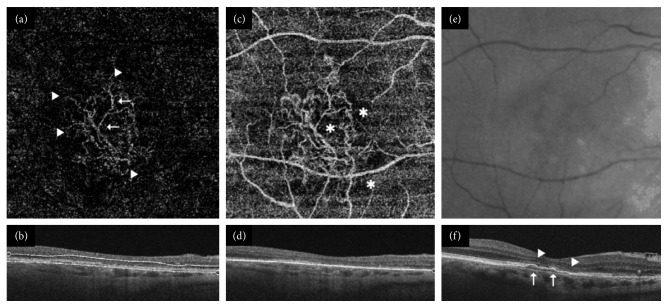
Clinically inactive hypodense choroidal neovascularization (CNV). (a) The swept source-optical coherence tomography angiography (SS-OCTA; DRI Triton, Topcon) en-face layer displayed filamentous well-defined vessels (arrows) with blunt endings (arrow-heads) in the absence of branching capillaries, intervascular anastomoses, and peripheral arcades in (b) an outer retina 4.5 × 4.5 mm segmentation image. (c) An underlying 4.5 × 4.5 mm choriocapillaris cube illustrated the CNV structure embedded in a dark nonvascularized halo (^*∗*^) with projection artefacts corresponding to the superficial retinal plexus of (d) the OCT B-scan slab. (e) An infrared picture of the quiescent macular region with isolated drusen but no signs of activity. (f) The spectral domain-OCT B-scan (Cirrus HD 4000, Zeiss) repeatedly demonstrated subfoveal flat irregular pigment epithelial detachment (arrows) without fluid in addition to disintegrated outer retinal architecture (arrow-heads).

If neovascular flow was detected, lesions were classified as follows:

1. Predominantly hyperdense if multiple branching capillaries, peripheral arcades, loops, and anastomoses predominated in >50% of the maximum lesion area depicted in the most suitable 2D en-face SS-OCTA segmentation image (Figure 1).
2. Minimally hyperdense if networks consistent of branching capillaries, anastomoses, and peripheral arcades were found in <50% of the lesion size (Figure 2).
3. Hypodense if filamentous well-defined vessels with blunt endings or ill-defined capillaries were depicted in otherwise nonvascularized halos (Figures 3 and 4).

A senior medical retina fellow independently surveyed and categorized the pattern distribution. An attending who was not affiliated with the study group was consulted for adjudication in case of disagreement or discordant assignment to one of the classes.

### 2.4. Statistics

Age, sex, and number of administered IVIs were drawn from patient charts. Univariate logistic regression analyses were applied to detect the impact of branching capillaries, anastomoses, peripheral arcades, well-defined vessels, nonvascularized halos, and vascular density on the probability of activity. All significant factors in the univariate analysis were re-evaluated in a multivariable logistic regression model. To account for the small number of events in no flow, Firth's penalized likelihood approach was used. Statistical analysis was performed using R Studio 1.0.136 and SAS 9.4. Figures were developed using Affinity Photo V1.5.2.

## 3. Results

Eighty-four eyes of 78 patients with a history of nAMD based on multimodal imaging met the inclusion criteria. Seven eyes were excluded from the analysis due to poor image quality. Hence, 77 eyes of 72 patients were enrolled in this study, 54 (70%) of whom were female. The mean age was 80.3 ± 8.2 (standard deviation) years. Laterality was distributed evenly (52% right eyes). The mean follow-up time was 5.4 ± 2.0 years after the onset of nAMD with an average of 20.2 ± 12.5 IVIs of anti-VEGF in total.

Fifty-eight (75%) clinically active eyes presented with fluid on SD-OCT B-scans or funduscopic evidence of new hemorrhage. Nineteen eyes (25%) remained clinically silent at the time of recruitment. CNV was depicted in 71 of 77 eyes (92%) by SS-OCTA. Fifty-four of 58 eyes (93%) with clinical signs of neovascular activity exhibited a definite CNV membrane on SS-OCTA. In addition, 17 of 19 silent eyes (90%) exhibited a CNV formation on SS-OCTA. No statistically significant relationship between the clinical status of nAMD and the detection rate of CNV on SS-OCTA could be observed (*p*=0.52). Sex (*p*=0.60), age (*p*=0.38), and laterality (*p*=0.38) did not have an impact on flow detection.

Twenty eyes (37%) with active and three eyes (18%) with inactive disease were characterized as predominantly hyperdense (Figure 1). Moreover, 20 (37%) clinically active and four (24%) clinically inactive eyes were described as minimally hyperdense (Figure 2). Fourteen eyes (26%) with active and ten eyes (59%) with inactive disease were labeled as hypodense (Figures 3 and 4). In the multivariate analysis, a borderline significant difference regarding clinical activity was found between predominantly hyperdense and hypodense lesions (*p*=0.05). No significant difference between the predominantly and minimally hyperdense (*p*=0.69) as well as the minimally hyperdense and hypodense (*p*=0.22) groups could be detected. Capillaries, anastomoses, and arcades were more frequently observed in all eyes independent of their clinical status, while major vessels and nonvascularized tissue were more often absent in all eyes. The same distribution was detected in eyes with active nAMD but not in quiescent nAMD as summarized in Table 1. Nevertheless, no parameter alone exhibited a significant relationship with the clinical activity of nAMD.

Sex (*p*=0.64), age (*p*=0.37), and laterality (*p*=0.95) had no significant impact on clinical activity.

## 4. Discussion

The relationship between the vascular density of CNV based on SS-OCTA en-face images and clinical signs of activity in nAMD after previous treatment with anti-VEGF was investigated and a borderline significant difference could be detected (*p*=0.05). Other descriptions of different CNV formations in OCTA and their incorporation into clinical practice have previously been investigated with varying results [12–14]. In concordance with the presence or absence of findings first described by Coscas in 2015, we attempted to develop a qualitative classification algorithm based on neovascular density as a predictive factor for clinical activity [11]. Herein, all employed characteristics were independently related to clinical signs of neovascular activity and none were significant (Table 1). However, criteria such as branching capillaries, end-to-end anastomoses, and peripheral arcades were continuously found in active disease and were more frequently missing in inactive eyes. Based on our findings regarding the distribution of smaller and more numerous vessels towards activity, a higher density could be assumed. Topcon's SS-OCTA device is currently not equipped with a quantification algorithm, which would divide intervascular planes into fractals, determine the area size, and count the numbers of fractals. Therefore, our classification concept was a qualitative assessment ideally similar to the well-established assessment used in conventional angiography known for decades. Independent reading centers were used to classify neovascularization based on the quality of dye distribution (e.g., early leakage and hot spot) within an en-face 2D imprint of the retinal and choroidal vasculature.

In our study, 37% (20/54) of predominantly hyperdense lesions were associated with active disease and 18% (3/17) with inactive disease. In addition, 20/54 (37%) active eyes showed minimal hyperdensity in contrast to 4/17 (24%) inactive eyes. The distribution of minimally hyperdense lesions indicated a low number of inactive eyes as the substantial reason for the lack of significance rather than a misinterpretation of data. A tendency towards significance (*p*=0.05) between the distribution of predominantly hyperdense and hypodense lesions was established. Nevertheless, the investigated SS-OCTA-based classification failed to adequately distinguish between activity status and hypodensity as 26% (14/54) with active and 59% (10/17) with inactive disease were labeled as hypodense. Hypodensity due to a hypothesized maturation of the vasculature could not be significantly related to inactive AMD [15]. Spaide described an alteration of the disease by targeting new vessel growth. Regression of the most recent vascular elements was possible with retreatment while more prominent vessels could not be eradicated and still exhibited activity to some extent. The discordance between hypodense patterns and nAMD activity requires further enhancement.

Grassniklaus and Green proposed a compensatory response of the CNV architecture as a recapitulation of the choriocapillaris perfusion in eyes compromised by nAMD [16]. They stated that main CNV vessels were present even when the disease was controlled and did not necessarily exacerbate the disease severity. In concordance with their findings, the CNV detection rate on SS-OCTA in this study was high (92%) in eyes complicated by nAMD subsequent to anti-VEGF treatment, independent of the clinical status. These data were consistent with those of other groups investigating the persistence of neovascular formation in inactive disease over time [17, 18]. In addition to well-defined vessels, nonvascularized halos were more often absent in both active and inactive diseases. These halos were part of the lesions and resembled areas of nonperfusion of the underlying choriocapillaris or detection loss due to obscuration [19]. The absence of one of the characteristics was not significantly related to inactivity. Similar to our definition of inactive CNV, “quiescent” CNV is a nonexudative form of nAMD that develops over time, and its growth is currently under debate as a predictive factor of activity or reduced retinal sensitivity [20]. Al-Sheikh et al. recently published their results on the qualitative and quantitative features of active and quiescent lesions on OCTA [21]. They compared the same biomarkers of active lesions before and after treatment to quiescent lesions and even quantified the pattern complexity by the fractal dimension. Roberts et al. differentiated the vascular features of CNV complexes based on a quantitative analysis (AngioTool) in two patient groups, which were divided only by their injection intervals (< or >6 weeks) and found no significance [22]. We used a similar approach to classify the vascular network complexity in a qualitative manner. No exact definition yet exists for eyes in remission. In our opinion, suspension of retreatment in previously active nAMD for ≥9 months indicated inactivity. Nevertheless, a mismatch still exists between hypodensity elaborated by SS-OCTA and the activity of displayed vessels *in vivo*, hypothecating that the new vasculature could still leak and take even longer to reach maturation.

Additionally, CNV structures were detected by different OCTA devices. Liang used the AngioVue SD-OCTA system (Optovue, Inc, Fremont, CA) operating at 70,000 A scans per s with 3 × 3 mm cubes in 2016 [14]. We previously investigated the detection rate of SS-OCTA in treatment-naïve nAMD and found it to be around 76% [7]. SS-OCTA's higher wavelength and A scan rate result in deeper penetration and better contrast of small neovascular structures. Novais et al. found significantly larger CNV areas on SS-OCTA images than on SD-OCTA, proposing a more accurate visualization of CNV [23].

The impact of subretinal fibrosis and atrophy on image quality and projection artefacts on OCTA targets a different problem [24]. Based on our research, these factors had no significant influence on the detection rate of CNV on SS-OCTA (geographic atrophy *p*=0.14; fibrosis *p*=0.88). Resegmentation of the corresponding layers in the affected eyes was frequent and crucial to differentiate blood flow due to neovascularity and choriocapillaris dropout within the choroidal vasculature.

This study has several limitations. The data were analyzed retrospectively. No endpoints for comparison of independently armed cohorts were proposed. Therefore, conclusions regarding the sensitivity or specificity of the tested device could not be drawn. Multiple graders would be sufficient to reproduce intergrader reliability for testing in a prospective study design. Another limitation was the qualitative characterization of CNV structures on SS-OCTA. A small number of eyes in remission were insufficient for the quantitative analysis of influencing factors and may lead to misinterpretation of morphological criteria in silent eyes. Only prototypes for quantification software currently exist and their value is yet to be confirmed in a reading center. For the most part, all attempts were analyzed using plane 2D segments, although it is well known that CNV growth occurs not only horizontally, but also vertically. It would therefore be appropriate to quantify vessel density in a predefined 3D space. However, no characteristics of CNV on OCTA have been standardized to date. To our knowledge, this is the first attempt to use OCTA-based morphological criteria for vascular density in a clinically applicable qualitative grading system.

## 5. Conclusions

In summary, the herein presented classification can partially replace current standard follow-up procedures. We were able to gain a better understanding of the development of the disease. Hyperdensity acts as an indicator of activity, but hypodensity does not necessarily reflect inactivity. The development of an objective qualitative and quantitative grading method is necessitated in a prospective study to confirm the reproducibility and reliability of SS-OCTA findings.

## Figures and Tables

**Table 1:** Presence of swept source-optical coherence tomography angiography-based morphological choroidal neovascularization characteristics in relation to clinical activity.

		Active nAMD *n* (%)	Quiescent nAMD *n* (%)	Total *n* (%)	*p* value
Branching capillaries	Present	37 (79)	10 (21)	47 (66)	0.45
	Absent	17 (71)	7 (29)	24 (34)	
	Total *n* (%)	54 (76)	17 (24)	71 (100)	

Anastomoses	Present	38 (83)	8 (17)	46 (70)	0.09
	Absent	16 (64)	9 (36)	25 (30)	
	Total *n* (%)	54 (76)	17 (24)	71 (100)	

Peripheral arcades	Present	32 (84)	6 (16)	38 (55)	0.12
	Absent	21 (68)	10 (32)	31 (45)	
	Total *n* (%)	53 (77)	16 (23)	69 (100)	

Well-defined vessels	Present	25 (81)	6 (19)	31 (44)	0.46
	Absent	29 (73)	11 (27)	40 (56)	
	Total *n* (%)	54 (76)	17 (24)	71 (100)	

Nonvascularized halos	Present	19 (83)	4 (17)	23 (32)	0.42
	Absent	35 (73)	11 (27)	46 (68)	
	Total *n* (%)	54 (78)	15 (22)	69 (100)	

nAMD = neovascular age-related macular degeneration.

## Data Availability

The datasets used and/or analyzed to support the findings of this study are available from the corresponding author on reasonable request.
